# Node of Ranvier as an Array of Bio-Nanoantennas for Infrared Communication in Nerve Tissue

**DOI:** 10.1038/s41598-017-18866-x

**Published:** 2018-01-11

**Authors:** Andrea Zangari, Davide Micheli, Roberta Galeazzi, Antonio Tozzi

**Affiliations:** 1 Azienda Ospedaliera San Camillo Forlanini, Pediatric Surgery and Urology Unit, Circonvallazione Gianicolense 87-00152, Roma, Italy; 2 TIM S.P.A., Wireless Access Engineering Department, Viale Parco de’ Medici, 61 - 00148 Roma, Italy; 3 Dipartimento di Scienze della Vita e dell’Ambiente, università Politecnica delle Marche, via Brecce Bianche, 60131 Ancona, Italy; 4 UOC Fisica Sanitaria, Azienda USL Toscana Sud Est, via Senese 161, 58100 Grosseto, Italy

## Abstract

Electromagnetic radiation, in the visible and infrared spectrum, is increasingly being investigated for its possible role in the most evolved brain capabilities. Beside experimental evidence of electromagnetic cellular interactions, the possibility of light propagation in the axon has been recently demonstrated using computational modelling, although an explanation of its source is still not completely understood. We studied electromagnetic radiation onset and propagation at optical frequencies in myelinated axons, under the assumption that ion channel currents in the node of Ranvier behave like an array of nanoantennas emitting in the wavelength range from 300 to 2500 nm. Our results suggest that the wavelengths below 1600 nm are most likely to propagate throughout myelinated segments. Therefore, a broad wavelength window exists where both generation and propagation could happen, which in turn raises the possibility that such a radiation may play some role in neurotransmission.

## Introduction

The intriguingly complex nature of the brain has always encouraged extensive studies on neuronal communication, aiming to understand signaling mechanisms and their integration into neural functions of the highest level. New perspectives have been revealed by approaching different biophysical mechanisms, which may coexist with the established chemical and electrical properties of cellular membranes^1^. In this context, previous studies on the electromagnetic properties of neurons gained increasing interest, resulting in further achievements and new open questions^2–4^. It seems therefore appropriate to explore the possible implications, which may add further knowledge to the current theoretical and experimental work in this direction.

Since early decisive studies, electrochemical phenomena have been shown to be predominant in the generation and traveling of information^5^.

The conduction of signals within neurons is sustained by a propagating phenomenon known as action potential (AP), which is a sharp change in the electrical potential across the cell membrane, in which different ionic species are involved. Once triggered, this process travels down the whole axon towards synapses. Some axons are coated with myelin, a multilayered lipid envelope, provided by surrounding glial cells and interrupted at regular distances. These gaps are called nodes of Ranvier (NR)^6^. In myelinated fibers the AP is triggered in the axon initial segment (AIS) and in the NR, where ion channels are concentrated, and leaps from node to node at a rate significantly higher than in unmyelinated axons. This process is known as “saltatory conduction”^7^.

The original Hodgkin–Huxley (HH) theory models each component of an excitable cell as an electrical element, taking into account the concentration of the main ionic species involved^5^. The transmission of APs in myelinated fibers has been described borrowing some concepts of the cable theory to simulate impulse initiation and saltatory propagation^8^.

Beside the fundamental mechanisms of neuronal membrane excitability described by the HH model, a number of other biophysical phenomena are associated with neuronal activity^1^.

Different physical approaches to these processes, which take into account mechanical forces, thermodynamics and electromagnetism, drew growing interest from researchers and may provide further understanding of the mechanisms underlying neuronal signaling and encoding of information^2,9^.

We focused our attention on the possible electromagnetic (EM) aspects of axonal impulse conduction, which have been investigated so far. Optical propagation of photons through myelinic waveguides has been recently shown to be possible by detailed modeling, and therefore raising the question of what could be the source of such radiation^4^.

Like any other cellular process, axonal activity involves energy generation and exchange. Since early investigations on neuronal function, measurements during action potential revealed the production of heat^10^, while infrared radiation transfer between nerve ends, following stimulation, has been experimentally detected^11^.

Beyond these reports, many researchers have been considering a possible role of EM radiation, either of the infrared or visible spectrum, in neural excitability and signaling, resulting in theoretical work on what has been referred to as an electromagnetic theory of neural communication^2^.

Actually, the existence and transport of infrared and visible light have been recently demonstrated in different tissues and even in nerves^3,12,13^.

Next to the studies on the existence of photon emissions as possible carriers of cellular information, different hypotheses of EM propagation through membranes or axonal structures have been advanced^14^, until recently, when a comprehensive model described the possible propagation of EM waves through optical communication pathways in the axon^4^. Alongside a growing interest in the interaction between EM radiations and biological tissues for its diagnostic and therapeutic implications, some evidence of axonal response to infrared and visible light has been observed, adding a further step towards an EM interpretation of neuronal signaling^15^.

However, as suggested by the aforementioned study on optical communication pathways in the axon, the possible sources of EM waves in certain cellular compartments have not yet been explored beyond speculative hypotheses and need further investigation^4^.

If we suppose that the propagation of action potentials in myelinated fibers occurs along axonal structures in the form of electromagnetic optical waves, then an explanation of their origin is needed. This should take place at the NR, where the action potential is regenerated. In particular, since electromagnetic radiation can be generated by charge movements, it could be hypothesized that its source relies on the flux of ions through channels in the time frame where action potential takes place.

Here we describe a model of generation of electromagnetic waves by active sodium channels at the sites where action potential is initiated or regenerated, as in the axon initial segment and in the NR of myelinated axons. A simulation of their propagation through axonal pathways behaving as waveguides is also provided, with findings in full agreement with those recently reported^4^.

## Results

### Electromagnetic wave propagation approach to the Node of Ranvier

In our model, we assume that electromagnetic waves arise at the axon initial segment and the NR, which act like a nanoantenna array system able to radiate optical waves propagating through the myelinated axon. Such a mechanism could in turn explain the so-called AP saltatory conduction by taking into account the electromagnetic wave propagation through the myelinated axon, as in an optical fiber^4^.

In particular, ionic currents through channels could be assumed as the sources of the electric currents pulses and behaving like a phased array of elementary dipole nanoantennas. An antenna array is a set of multiple connected antennas, which work together as a single antenna, to transmit or receive electromagnetic waves^16^. Each individual antenna element is fed in a specific phase relationship with respect to the others. Waves radiated by each individual antenna combine and superpose, adding together (interfering constructively) to enhance the power radiated in desired directions, and cancelling (interfering destructively) to reduce the power radiated in other directions. An antenna array can achieve higher gain (directivity), which results in a narrower beam of EM waves, than it could be achieved by a single antenna. In general, the larger the number of individual antenna elements used, the higher the directivity gain and the narrower the beam^17^. The nanoantenna array here considered for numerical analysis consists in a certain number of dipole antennas distributed and located in several planes sequentially placed, each one representing a section of a cylindrical node segment. The thickness of each section corresponds to the linear sodium channels distribution along the node. In particular, the number of antenna elements per unit length corresponding to the sodium channels density, was chosen within a range of measured values pertaining to the rat optic nerve. Some literature^18^ reports a channels density of 1400/um^2^, measured by a freeze-fracture study on the rat optic nerve, from which a 27 nm frame distance of antenna planes can be inferred. However, few papers provide estimates of channel densities of single species and fiber types and these may vary with measurement methods^18,19^. Therefore, in order to evaluate different configurations, we utilized a 27–50 nm distance range, inclusive of 400–700/µm^2^ channel density values from a saxitoxin binding study in a similar rodent species: the rabbit^19^ (Table 1). Due to the amount of data to be computationally processed when considering the entire node or axon initial segment length, in the range 0.7–1.4 µm^20^ and 10–60 μm respectively^21^, only a limited segment has been numerically simulated. The diameter, which can be approximated to that reported for the axon, has been set at 0.5 µm. This value falls within the 0.27–3.12 µm interval calculated from the g-ratio of 0.78 (axon diameter/fiber diameter) and the fiber diameter of 0.35–4.0 µm for the rat optic nerve (Table 1)^22–25^.

**Table 1:** Main structural features of Rat optic nerve **(**values taken from the references within brackets).

Rat optic nerve features	Main values
Fiber diameter	0.35–4.0 µm^24^
g ratio	0.78^23^
Axon/Node diameter*	0.27–3.12 µm (calculated from fiber diameter and g ratio)
	0.80 µm^20^
	0.77 µm^21^
Myelin thickness	0.13 µm (65 days old rat)^22^
Nodelength	1.08 µm (0.7–1.4 µm)^20^
	0.8 µm^21^
Node sodium channels density	1400/μm^2^ ^18^
	400–700/um^2,^* ^19^
Node calculated distance between channels	27 nm (37 ch/um)
	38 nm (26 ch/um)*
	50 nm (20 ch/um)*
	*Rabbit optic nerve^19^

Finite element method (FEM) numerical analysis of the electromagnetic propagation is computed by COMSOL Multiphysics commercial software.

The numerical simulation of NR-equivalent nanoantenna array system shows that when each antenna plane section is time triggered with appropriate phase delays, the main radiation pattern of array is directed towards the subsequent internode, i.e. the dielectric waveguide, where the energy is propagated up to the next node. This is a quite interesting result, as the energy turns out to propagate mainly in the useful direction rather than diffuse at random in the surrounding medium. This further supports the hypothesis of the core-shell fiber optic structure for the EM wave propagation in the myelinated axon^4^. Moreover, it is worth of mention that nanoantennas can really exist and work only in the time interval when ion currents flow, thus preventing spurious phenomena.

### Full wave simulations of forty λ/100 nanodipoles antenna array

The present section deals with the simulation of an array of 40 dipoles. The array consists of 5 planes each containing 8 dipole antennas, symmetrically placed around a cylinder of radius 0.5 µm. The membrane resting potential (V_m_) and the sodium equilibrium potential (V_eq_) in the small caliber fibers of the rat optic nerve considered in the study are −72 mV and 48 mV respectively^26^. The potential of the driving force (V_df_), by which ions cross the membrane during the AP, is given by V_df_ = V_m_ − V_eq_ and it amounts to 120 mV at the beginning of the AP, then it reduces to 0 mV at the peak. In this full wave simulation, the voltage supply at the antenna ports has been set to 100 mV, which corresponds to V_df_ = −100 mV and V_m_ = −52 mV. The feed phase delay time between antenna planes is in the range from 0 to few μs. Each plane of nanoantennas is placed at 36 nm distance from the previous one, a value which falls in the interval between 27 and 50 nm, as mentioned above. The case of 36 nm corresponds to sodium channels density of 700/μm^2^. In these simulations, the wavelengths are in the range 300–2500 nm. The length of each dipole antenna has been chosen to match the length of sodium channels i.e. approximately 13 nm^27^.

Even with this arrangement, limited to 8 current sources for each section, the power is radiated mainly in the useful direction. Nevertheless, the model matches the real node channel density^18,28^ and it is only slightly shorter than a 0.7–1.4 µm long real node^20–22^, thus allowing the simulation of a considerable portion of it.

**Figure 1:**
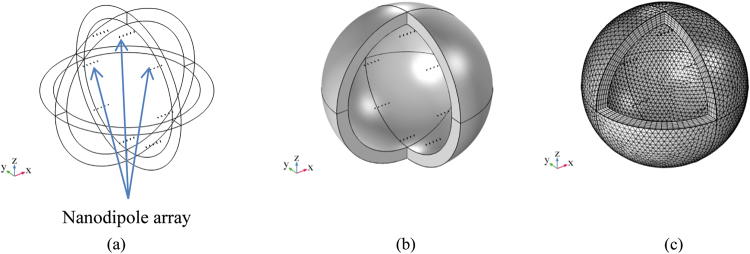
FEM simulation of nanodipole array assuming (**a**,**b**) geometry and (**c**) mesh of the 40 dipole antennas.

Nanoantennas have the dimension in the range λ/50, λ/100. In^29^, the case of λ/50 antenna is studied. In particular, it is shown that the dipole radiated power amplitude pattern has only a slightly lower gain in directivity compared to the standard λ/4 antenna (Fig. 1).

**Figure 2:**
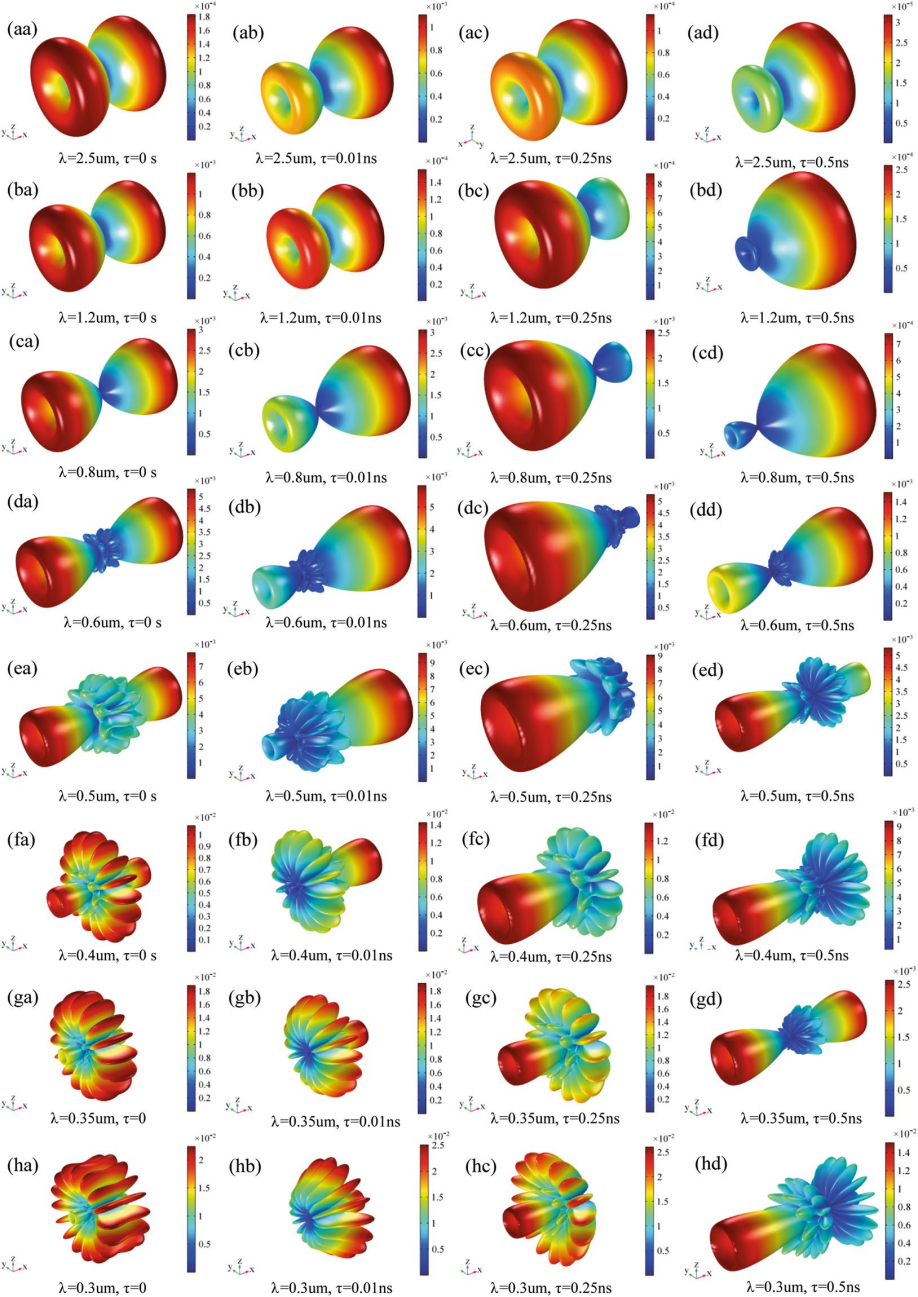
3D pattern of electric far field V/m for λ in the interval 0.3–2.5 μm and τ in 0–0.5 ns.

**Figure 3:**
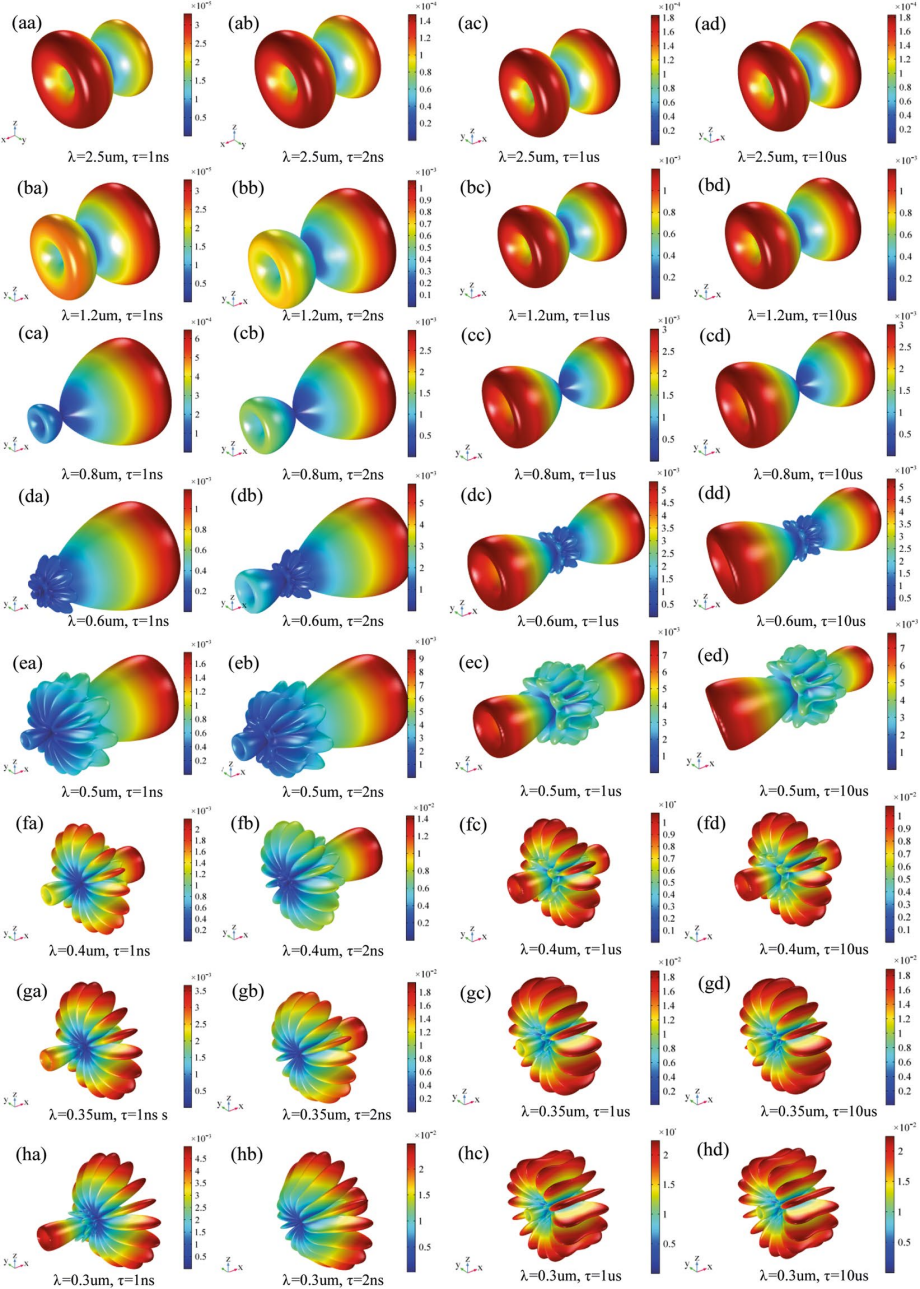
3D pattern of electric far field V/m for λ in the interval 0.3–2.5 μm and τ in 1ns-10 μs.

Beside, Figs 2 and 3 show the 3D electric far field pattern for different wavelength values in the range 300–2500 nm and different antenna port delay time to trigger ranging from 0 to 10 μs. Two interesting facts are worth of mention. Firstly, the higher the wavelength, the more focused the radiation patterns. This means that nanoantenna array configuration works better in the infrared portion of the wavelength interval i.e. above 700 nm. On the contrary, the lower the wavelength, the higher the side lobes of array. Such side lobes represent energy, which is lost as it propagates away from myelinated axon. Secondly, when time delay is 0s the pattern lobes show a symmetrical configuration, whereas for time delay greater than 0s one of the main pattern lobes enhance the array gain towards one preferential direction. Not all time delays reported in Figs 2 and 3 prove to be effective for a propagation in the forward direction; in some cases, we observe a back propagation and we hypothesize that only some values of time delay or phase shift are allowed. One interesting consequence of the aforementioned phenomena is the capability of the *node-myelin* system in regenerating and propagating the information in one preferential direction along the nerve structure. Nonetheless, the myelinated axon seems capable to radiate a large portion of the optical wavelength spectrum, thus providing, in principle, a large channel capacity.

### Electromagnetic wave propagation approach to the myelinated axon

#### Rat Optic Nerve like equivalent dielectric waveguide at optic wavelengths

**Figure 4:**
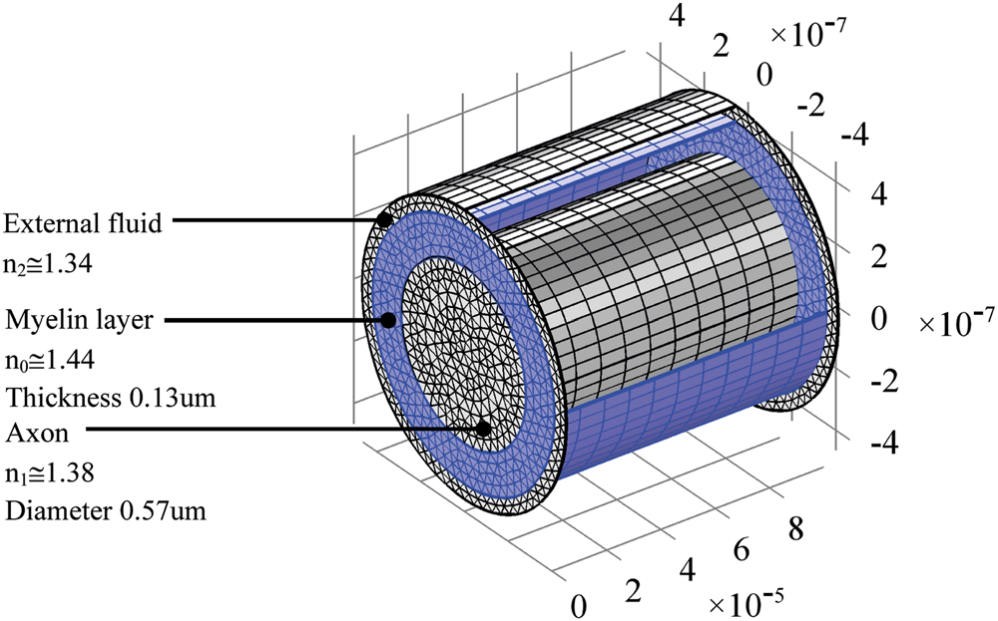
Scheme and mesh of rat optic nerve structure. Axes dimension are in m.

Propagation at a given wavelength can occur only via certain discrete modes, each with unique transverse field configurations and axial wave numbers. The configuration of myelinated axon is reminiscent of an optical fiber. In particular, the optical fiber core is typically the region where the most of energy propagation takes place (typically 50 µm in diameter for multimode propagation, while 5 µm diameters are typical of single mode fiber). Typical optical fiber consists of a cylindrical core with index of refraction n_1_, surrounded by a cladding with a refraction index n_0_ < n_1_. In myelinated axon here considered, the configuration is different; the core typically shows lower values of refraction index with respect to the myelin-surrounding layer. In Fig. 4, the mesh of the rat nerve is shown.

**Figure 5:**
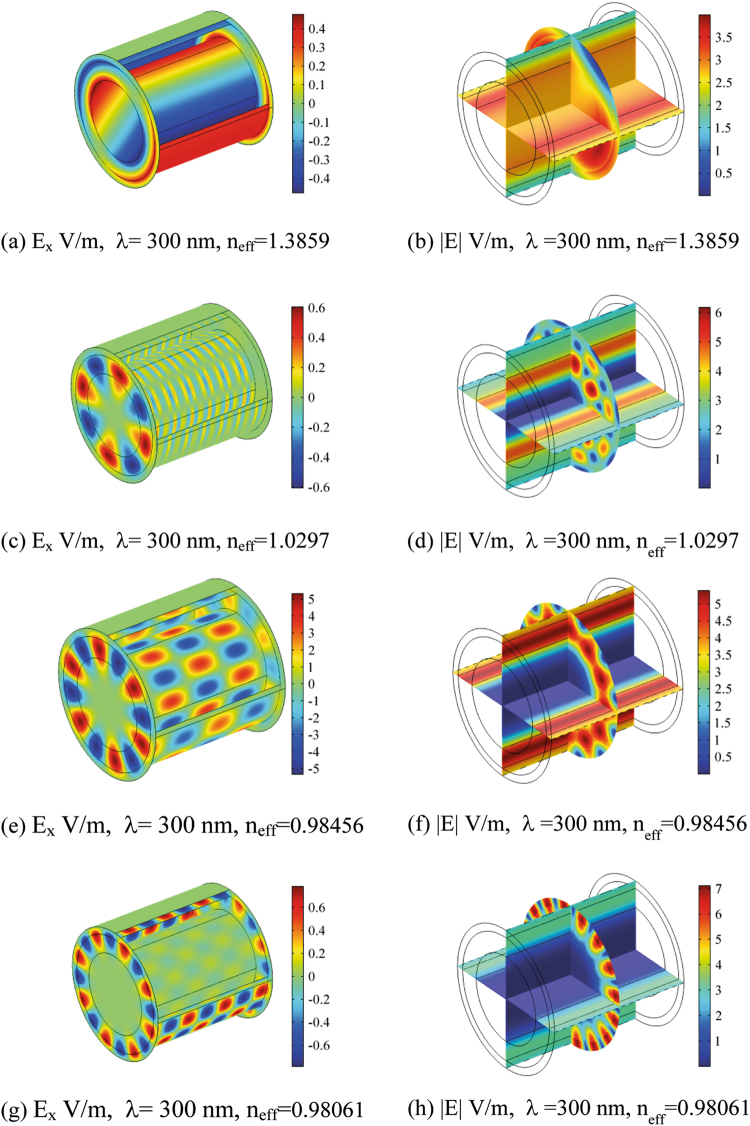
Electric field component E_x_ and Electric field norm distribution along the rat nerve structure at 300 nm wavelength for different refraction effective index values, n_eff_.

**Figure 6:**
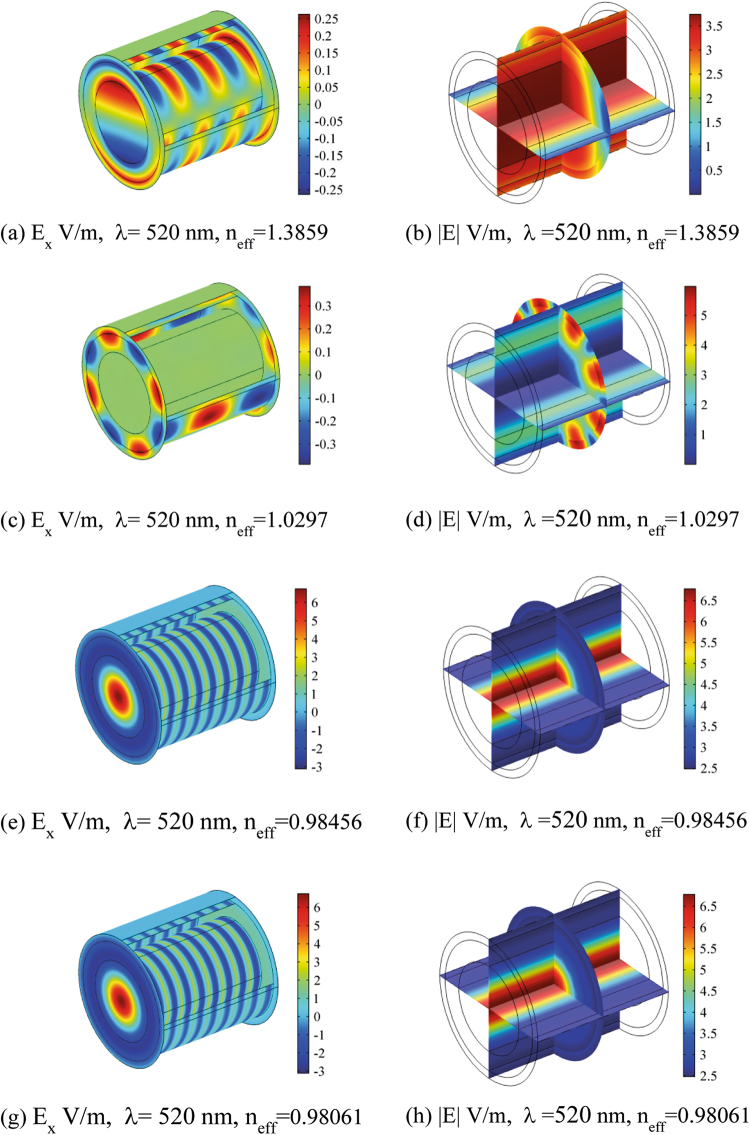
Electric field component E_x_ and Electric field norm distribution along the rat nerve structure at 520 nm wavelength for different refraction effective index values, n_eff_.

**Figure 7:**
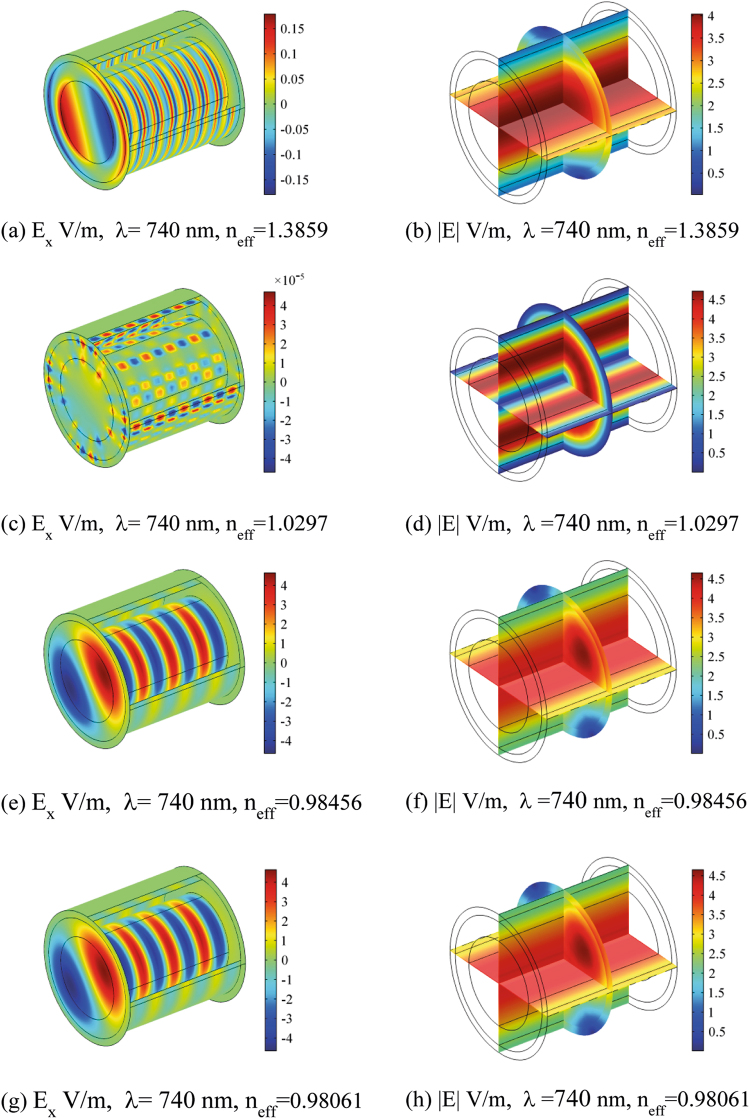
Electric field component E_x_ and Electric field norm distribution along the rat nerve structure at 740 nm wavelength for different refraction effective index values, n_eff_.

**Figure 8:**
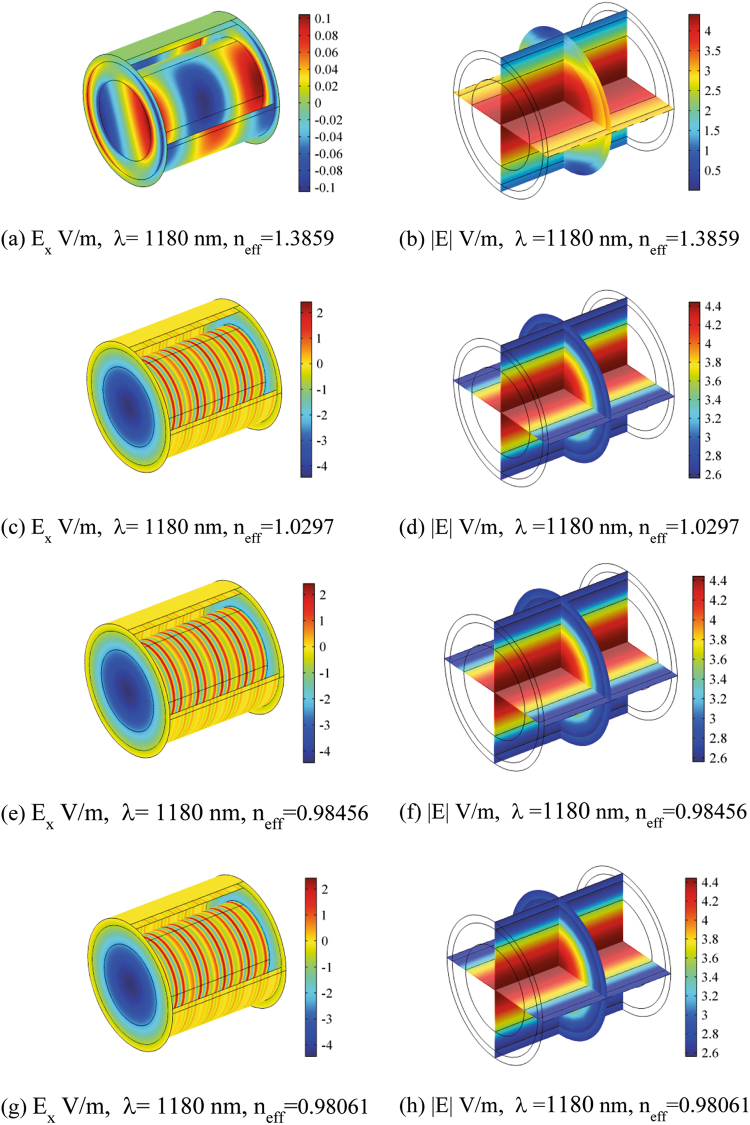
Electric field component E_x_ and Electric field norm distribution along the rat nerve structure at 1180 nm wavelength for different refraction effective index values, n_eff_.

Figures 5, 6, 7 and 8 show the electric field distribution at different wavelengths of 300 nm, 520 nm, 740 nm and 1180 nm and for all effective propagation indexes reported above. Moreover, in Figs 6 and 7 the wavelengths are in the visible spectrum whereas in Fig. 8 wavelengths are in the infrared spectrum. Above 1800 nm, the propagation ability for such waveguide geometry is reduced and above 2100 nm no propagation takes place. The refraction indexes used in this simulation are taken from^4^. Observing the reported plots of electric far field radiated by the nanoantennas array in Figs 2 and 3, the minimum amount of secondary radiation lobes takes place above wavelengths around 600–700 nm. Consequently the most probable wavelength spectrum of propagation should fall in the interval 700–1800 nm. Taking into account the absorption effects above 1600 nm^4,30^, the useful bandwidth probably reduces to 700–1600 nm. In addition, some modes mostly propagate into the axon whereas others do best in the myelinated layer, so allowing multiple mechanisms of energy propagation as a function of wavelength.

The emitting sources are simulated as numerical ports with a power of 10^−14^ (W). The first modes (those with the largest effective mode index) are shown. The plots show both the E_x_ component of electric field and the norm of electric field in V/m (Figs 5–8). The color legends can have different scales and the fields can show either minima (in blue) or maxima (in red) as a function of the propagation mode^31^.

### Electromagnetic field analysis of the full structure of the node of Ranvier coupled to the myelinated axon through a paranode section

#### Full electromagnetic wave numerical simulation

**Figure 9:**
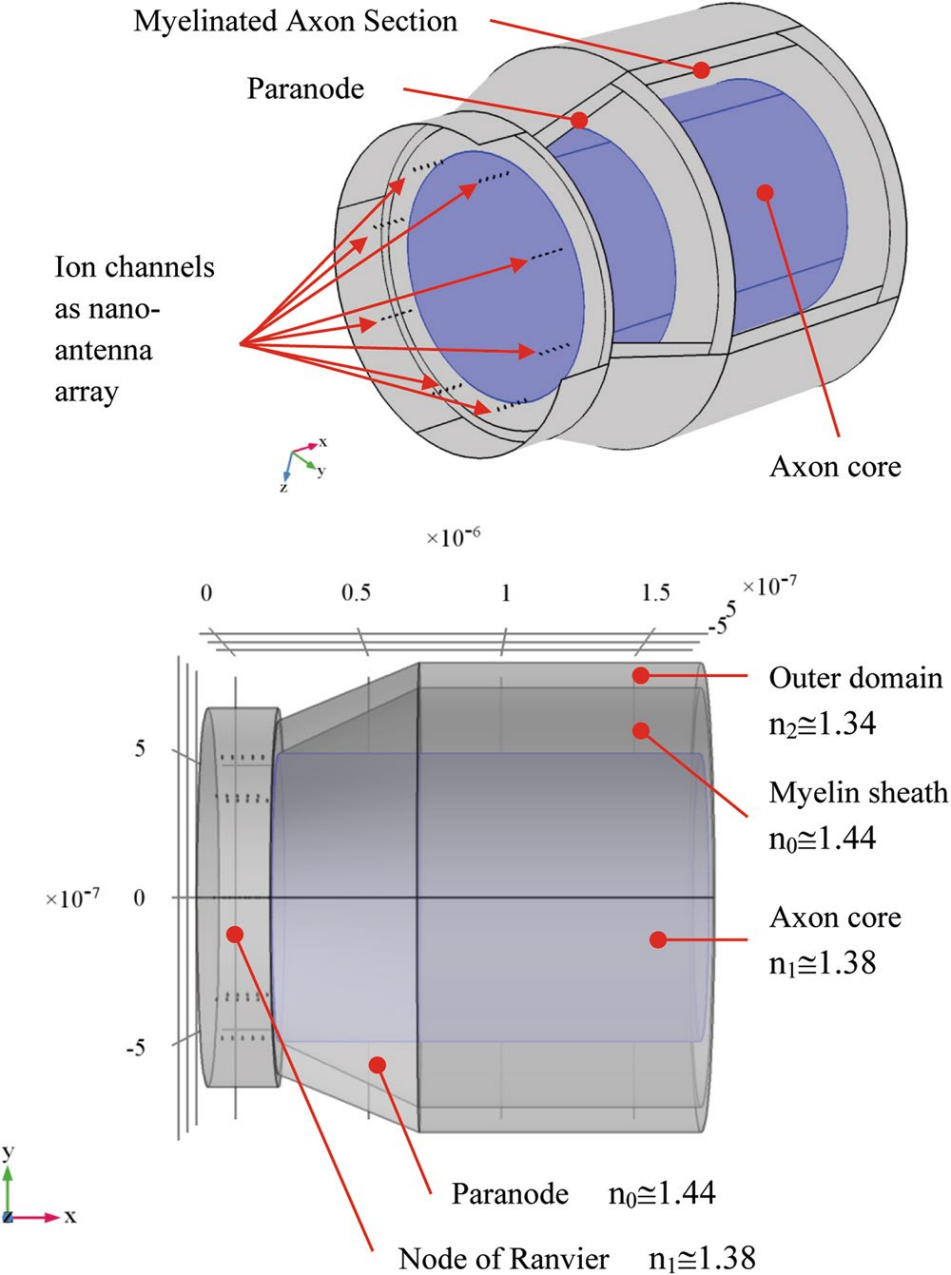
Geometry of the node of Ranvier coupled to the myelinated axon through a paranode section. In figure the dimension in m are shown.

In order to analyze the electromagnetic behavior of the full structure formed by the NR and the myelinated axon coupled by means of a paranode medium, a full wave numerical simulation has been performed. The geometry of the structure is shown in Fig. 9. where a 3D view and a 2D front view are displayed. In Fig. 9 the nanoantenna array (ion channels) are located in the NR.

The morphology and dimensions of the NR remain the same of previous simulations, the nanoantenna port voltage excitation as well. A paranode annular volume has been added around the axon core to provide an interface coupling the NR to the myelinated axon. The length of annular paranode part was set to 0.5 μm. This is the approximate length of the paranodal region where cytoplasm-rich paranodal processes turn into increasingly thick and compact myelin. For simplicity, the paranode has been shaped like a truncated cone. The cylindrical core of the paranode has the same shape and refraction index of the axon, whereas the external annular part has the refraction index of the myelin sheath. The axon core diameter is about 1.026 μm, the thickness of myelin layer is 0.234 μm and the total fiber diameter is 1.674 μm.

**Figure 10:**
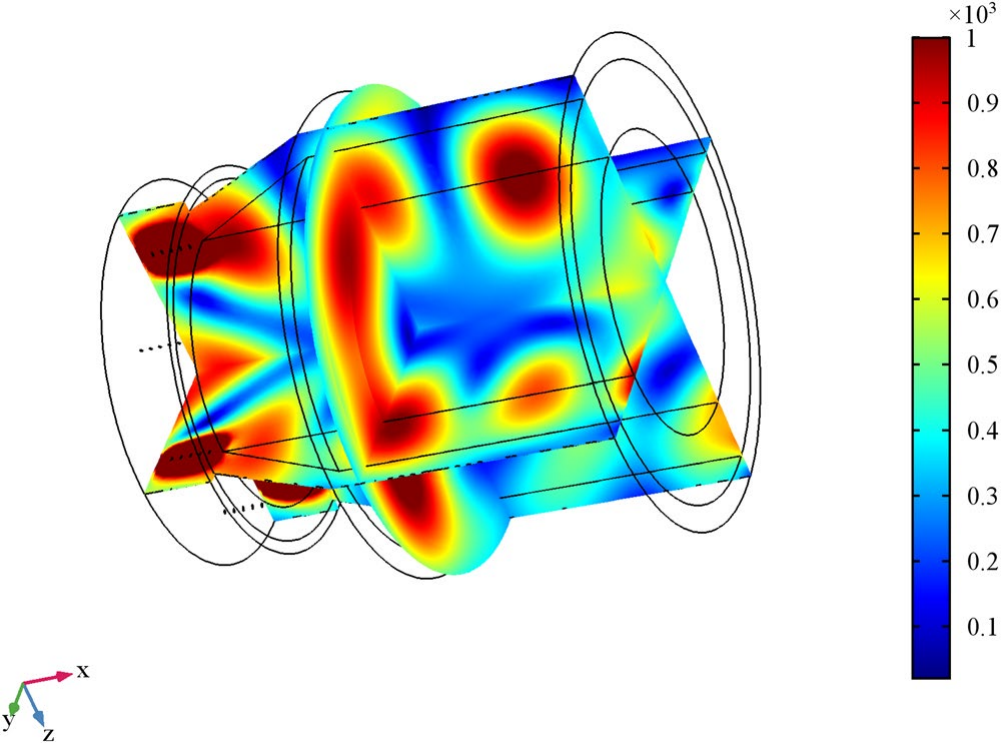
Full electromagnetic wave simulation for λ = 1200 nm. Electric field norm V/m.

**Figure 11:**
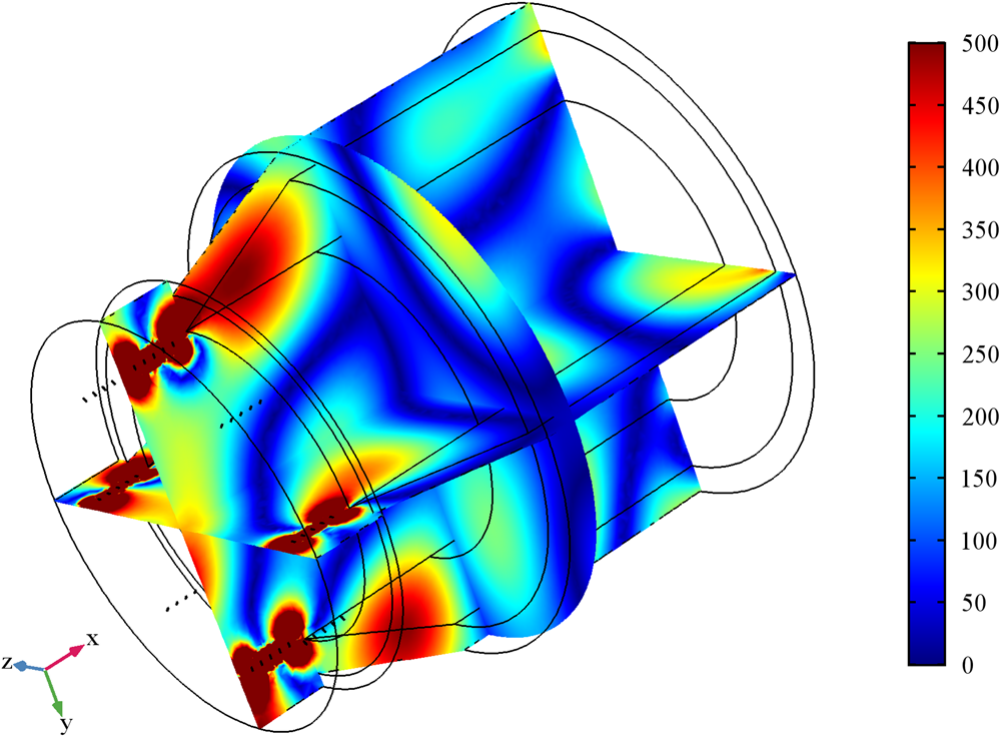
Full electromagnetic wave simulation for λ = 1200 nm. Electric field Ex component along x axes. Norm value expressed in V/m.

Some interesting findings are worth looking at. First of all, the space surrounding the nanoantenna array shows the highest value of electric field as expected. In fact, the array is the place where the EM field is generated by ionic currents, as explained in previous sections. Moreover, the geometrical configuration of the electromagnetic field in Figs 10 and 11 shows a preferential field propagation toward the myelinated layer through the paranode segment. The paranode seems to match the wave impedance of the NR site to the wave impedance of the myelinated axon. It works like a transformer, which electromagnetically optimizes the wave impedance to match the two different mediums. In the absence of such impedance matching layer, part of the radiated power could be reflected back to the antenna, so badly affecting the energy transmission efficiency and finally reducing the signal to noise ratio through the propagation channel.

This property of the paranode seems confirmed by the electric field distribution in the myelinated axon. Indeed, the maximum value of electric field is focused on the myelin layer where most of the wave propagation takes place. Even though the length of myelinated axon is limited to few microns due to computational constraints, the wave propagation is well observable by the periodic distribution of maximum and minimum values of the electric field norm along the myelinated axon reported in Fig. 10. In Fig.11 the norm of the component of the electric field E_x_ along x axes of propagation is plot. In this figure, the role played by the paranode in the forward electromagnetic field propagation can be observed.

## Discussion

Evidence that cells are able to generate and detect electromagnetic fields (EMF) has been accumulating since the early 20^th^ century. As stated by a comprehensive review, a multitude of biological effects have been experimentally investigated and attributed to a broad spectrum of wavelengths^32^. Spontaneous emission of light from cells is usually referred to as ultra weak photon emission (UPE) or biological luminescence. Cells usually emit UPEs with increased intensity when they undergo physiological changes, especially when they are exposed to chemical or physical stressors, whereas the association between UPE and reactive oxygen species is well known^13,32–34^. However, since photon emission and propagation in the proposed model occur in a spatially oriented constraint, represented by the node-myelin axis, such photons are unlikely to be detected in the environment, even in close proximity of the cell; therefore, they probably do not participate to what is currently known as UPE^35^. The occurrence of electromagnetic phenomena in electrically active cells and their relationship with cellular function have been investigated in the heart tissue and in neurons^36^. In this scenario, where electromagnetic (EM) effects seem to be widespread in a variety of biological systems, neural system is not an exception and such phenomena are increasingly addressed to single aspects of neuronal function. Yet, an electromagnetic interpretation of the whole sequence of neuronal signaling is incomplete.

**Figure 12:**
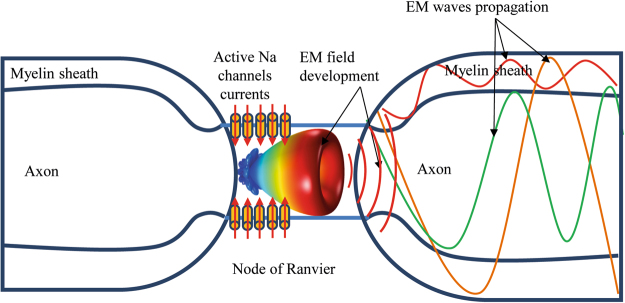
Schematic representation of ion channels in a Node of Ranvier behaving as nanoantenna array generating EM radiation.

We showed that ion channels in a node of Ranvier are reminiscent of a nanoantenna array and, in the assumed conditions, they behave likewise, being able to generate EM radiation in the spectrum of visible up to the infrared wavelength (Fig. 12).

Some aspects highlighted by our model and their relationship with current knowledge on the action potential are worth to be addressed here:

1. It is conceivable that electrochemical and electromagnetic mechanisms of neural signaling in the axonal compartment are not in contradiction; rather they are strictly associated phenomena, sharing a common origin, which relies on ionic channels activity. Because of neuronal activity in the brain, transmembrane currents, including ionic fluxes through channels, are known to contribute to electric and magnetic fields, which can be recorded in the extracellular medium^37,38^.
  Based on isolated neuron models, electrical response of neurons to noise-like electromagnetic radiation has been studied as a possible mechanism of mode selection and transition of electrical activities in neurons exposed to electromagnetic radiation^39,40^. The implications of such a field activity of neurons have been investigated by modeling and interpreted as a possible form of cellular and intercellular distant communication. Such studies suggest that the contribution of local components of these phenomena, such as ionic channels activity at the node, is worth to be considered^41,42^.
2. The source of EM signals is thus set at the node, where it is expected to be, given the axon optical waveguide properties and the hypothesized propagation between myelin ends^2,4^.
3. The EM directional radiation pattern analyzed in our model reflects the typical polarity of neurons, featured by the unidirectional propagation of the action potential^43^.
4. The role of ionic channels in signal modulation as established by electrochemical studies is not undermined, rather it is strengthened by the much higher flexibility and modulating capabilities of the electromagnetic spectrum with respect to information encoding and transfer.
5. Our simulation does not fulfill a general mechanism, rather a local one, as an evidence of extreme specialization of the many different electromagnetic phenomena, which have extensively been demonstrated in biological systems. Herein it corroborates the concept of a possible evolutionary optimum, by which energy byproducts, such as photons, integrate existing mechanisms^15^. From an evolutionary standpoint, among the essential requirements of a given mechanism to be advantageous in terms of information processing and transmission are energy saving, conduction speed and signal clearness. Some of the primary advantages of myelin are, undoubtedly, improvement of conduction velocity and energy saving, by reducing the number of channels employed. Such evolutionary useful features of myelin are shared with a hypothetical mechanism of photonic energy transport and further highlighted by their reciprocal functional relationship occurring in the optical waveguide hypothesis. Whilst the mechanism we described satisfies such requirements (higher conduction velocity and lower energy expense), the need of signal clearness by noise reduction is also accomplished. It has been advocated that UPE activity is unlikely to be a widespread mechanism of cell-to-cell communication due to noise effects, given the multitude of cellular processes able to produce a variety of UPE^35^. In our simulated model, photons are generated only when ionic currents assume a certain precise space-time configuration, thus reducing the effects of spurious signals. In fact, in the absence of certain space-time currents’ relationships, the radiation mechanisms do not take place, thus intrinsically avoiding leakages and spurious propagation phenomena. In the achievement of the most complex brain functions, such an evolutionary significance of photonic energy is impressively addressed by recent experimental evidence: photonic activity induced by glutamate in the brain shows a wavelength shift towards the red, in a phylogenetic scale from frogs to humans. Since longer wavelengths carry lower energy, the involvement of photons in neural communication processes well suits the general principle by which increasing complexity of functions requires better energy expense optimization^44^. Interestingly, the reported spectral interval partially overlaps with the range of wavelengths, which are most likely to originate and propagate in the myelinated axon, as issued by our findings and other previous studies^4^.
6. Whether and how environmental noise caused by natural light, may affect our proposed propagation mechanism, is worth of discussion.
  Myelin sheath has been a crucial acquisition of vertebrates. One of its advantages is an increased speed of conduction to adapt to larger body sizes, without increasing axon diameter. Another advantage is the adaptation to the space constraint of skull and vertebrae^45^. Meanwhile, new apparatuses, with protective functions from the external environment were achieved by the evolution of this subphylum, such as the skeleton and the skin, with its pigments and integumentary adnexa. These represent a relevant barrier to environmental physical agents, including light. The effective amount of light reaching the finest axonal structures, such as nodes, should be considerably reduced, in the central nervous system by the skeletal system and in peripheral nerves by the skin and integumentary organs. However, despite the existence of such barriers, possible influence of noise due to natural light cannot be excluded and will be farther examined.
  A major obstacle to electromagnetic signal detection by cells in natural conditions is an unfavorable signal-to-noise ratio^35^. A number of principles have been described to show how cells could achieve a more favorable signal-to-noise ratio. A possible solution to limit the influence of environmental noise is the existence of special propagation pathways, which is well suited by the node-myelin complex, where the emitted signal is oriented towards and confined to the myelinic optical guide^35^. Indeed, it has been argued that allowing light from a defined direction to reach the detector might result in substantial noise suppression. In our model, such a spatial filtering mechanism is achieved through the oriented constraint of emission and the optical boundaries of the signal. These principles refer to communication over intercellular distances^13^. In our model, signaling occurs in an intracellular domain and between cellular structures, thus in much shorter distances. This is an advantage in achieving a spatial alignment between the transmitter and the receiver, which is another requirement for a favorable signal to noise ratio^35^. Since the intensity of radiation involved in the described model is unknown, an estimate of signal to noise ratio is not available. Due to the extremely circumscribed space bound in which it occurs, new experimental techniques are needed to detect and quantify photons involved in the process. Such methods of detection may be offered by nanotechnology, for example by the use of nanoparticles to be delivered at the node and able to locally detect photons^46^.
7. The intracellular medium, or axonal cytoplasm, is rich in organelles and different proteic structures, such as microtubules. The computed absorption spectrum of the microtubules in the low-frequency THz regime has been investigated by large scale molecular dynamics simulations^47^. Unfortunately, these results cannot be generalized in a straightforward way to our much shorter wavelength regime, because even though the chosen mean refractive index of cytoplasm would allow the propagation of photons, the microscopic index is expected to vary wildly over a distance of one wavelength. Further modeling and experimental evidence are needed to investigate the interaction of photons involved in the described model with microtubules and other intracellular structures.

Despite our model adds a further step to the sequence of possible axonal propagation of EM waves, offering a model of its source, hypotheses on how this signal could promote a new action potential at the next node are still lacking.

As a matter of fact, experimental evidence has been achieved that infrared radiation is able to activate action potentials, as a result of thermal effects by reducing the membrane capacitance^12,48^. This in turn has been ascribed to a process of membrane nanoporation, resulting in sodium inflow and ultimately in a voltage dependent response of channels^49^.

Another possibility to explore involves a direct influence of the radiation on channel gating. A number of new achievements on channel proteins structure and function provide promising suggestions.

In fact, it is well known that nerve cell membranes are equipped with a variety of voltage-gated ion channels (VGIC), whose function is to regulate the frequency and pattern of the AP. Moreover, ion diffusion can be promoted by cells by the cooperation of different types of receptors, such as ligand-gated, mechanically-gated, voltage-gated and finally light-gated or temperature-gated ion channels.

Indeed, among the latter category, new evidence has appeared that, in addition to membrane potential, some members of the VGIC superfamily are responsive to other physical and chemical stimuli, sometimes involving additional regulatory domains and that different gating mechanisms can coexist in various types of ion channels^50,51^. Among these regulatory domains, a central role is gained by Per-ARNT-Sim (PAS) motifs, firstly identified as homologous regions (about 50 aa) in the proteins Per, ARNT and Sim, and representing a widespread component of signal transduction proteins. Indeed, this kind of domain plays the role of universal signal sensor and it is able to detect a wide variety of physical and chemical stimuli such as temperature variation or light^52^. Moreover, Tang *et al*. resolved the crystallographic structure of the PAS domain of a potassium channel (hEAG, Homo sapiens EAG-related gene; pdb code 5j7e), that is involved also in neuronal excitability^53^ thus suggesting that it can be also related to other VGIC family members.

Protein channels found in sensory neurons in the skin and mucous membranes are here worth of mentioning since either an increase in temperature or decrease in temperature or other mechanical stimuli are able to open the channel thus allowing ion permeation.

For instance, this polymodal behavior has been demonstrated in transient receptor potential (TRP) channel family members as the Painless or TrpA1 channels, which are responsive to ligand, mechanical stretching and heat^54^. In the hypothesis that the energy release to the channel is temperature-mediated, it is striking that the mechanisms underlying temperature sensitive properties of TRPs occur in many VGIC superfamily members, due to helical bundle domains bearing buried polar residues proximal to the pore^55^.

In fact, the full knowledge about eukaryotic voltage-gated sodium channels (Na_v_) gating is still lacking and many informations arise from bacterial sodium channels (BacNa_v_) conduction and activation, as they represent the prokaryotic sodium channel orthologues. As a result, recently, a BacNa_v_ cytoplasmic domain is found to control channel gating through a temperature-dependent reversible structural transition in a metastable domain proximal to the ions’ pore^55,56^. Besides, the first complete structure of Na_v_ in the activated open form has been resolved^57^. This elucidated structure represents a starting point for dynamical simulations of ion translocation since it enables the visualization of new interactions between all the channels’ domains. Further research should point to uncover new sites or the possible role of all the Na_v_ channel domains, whose response to photonic energy may end in channel opening.

A question may arise whether the energy carried by the propagating EM wave would be quantitatively in the range of the free energy of activation of a voltage-gated sodium channel^58^, either in the order of ion permeation’s energy barriers^59^ or in sufficient amount to operate a nanoporation as the initial event of voltage gated channel activation^49^. Further investigation is needed to better characterize the energy output across the optical guiding system and whether this would be able to cooperate with or even initiate the whole process currently known as action potential.

Despite many questions are still unanswered, the exposed argumentations raise reasonable suggestion that our approach, together with the optical guiding system hypothesis, not only is consistent with a possible involvement of photons in neuronal signaling, but also it represents a contribution to disclose their possible role in integrating other known mechanisms for information transmission in the myelinated axon. In the light of current knowledge, photonic activity in the spectrum of light and infrared does exist and it is transmitted in evolved animal neural circuits. We think that the involvement of photonic energy in the most complex brain functions has reached by now large evidence, culminating in the striking demonstration of its evolutionary role^44^ and thus embodying the principle, called natural selection, by which each slight variation, if useful, is preserved^60^.

## Methods

### Introduction to radiation pattern by four λ/4 microdipole antenna array

In the results section we reported the array of forty nanoantennas model analyzing the infrared electromagnetic propagation. In order to develop this configuration, we started our analysis from the most elementary classic λ/4 dipole antenna configuration.

In this simple λ/4 dipole antenna model, an antenna array is made of four microdipoles cross placed with entering currents. In particular, the electric far field of antennas array is computed by means of FEM analysis technique using COMSOL Multiphysics commercial code^61^. The electromagnetic waves, frequency domain physics interface, is used to solve for time-harmonic electromagnetic field distributions. For this physics interface, the maximum mesh element size was limited to a fraction of the wavelength. The domain size that can be simulated thus scales with the amount of available computer memory. A perfectly matched surrounding layer has been added before defining the far-field domain. The electromagnetic waves, scattered field, frequency domain analysis, was applied to solve the equation and to find values of the electric field E expressed in V/m:

1 $$\nabla \times {\mu }_{r}^{-1}(\nabla \times {\bf{E}})-{k}_{0}^{2}({\varepsilon }_{r}-\frac{j\sigma }{\omega {\varepsilon }_{0}}){\bf{E}}=0$$

where

ε_r_ is the real part of the relative electric permittivity;

ε_0_ is the electric permittivity of vacuum;

μ_r_ is the real part of the relative magnetic permeability and is equal to 1 since no magnetic material is considered here;

σ is the electric conductivity of antenna elements set in the range 0.01–0.08 S/m and computed by means of Conductance around 20 pS^62^, and by the dimension of channel applied in the simulation.

**Figure 13:**
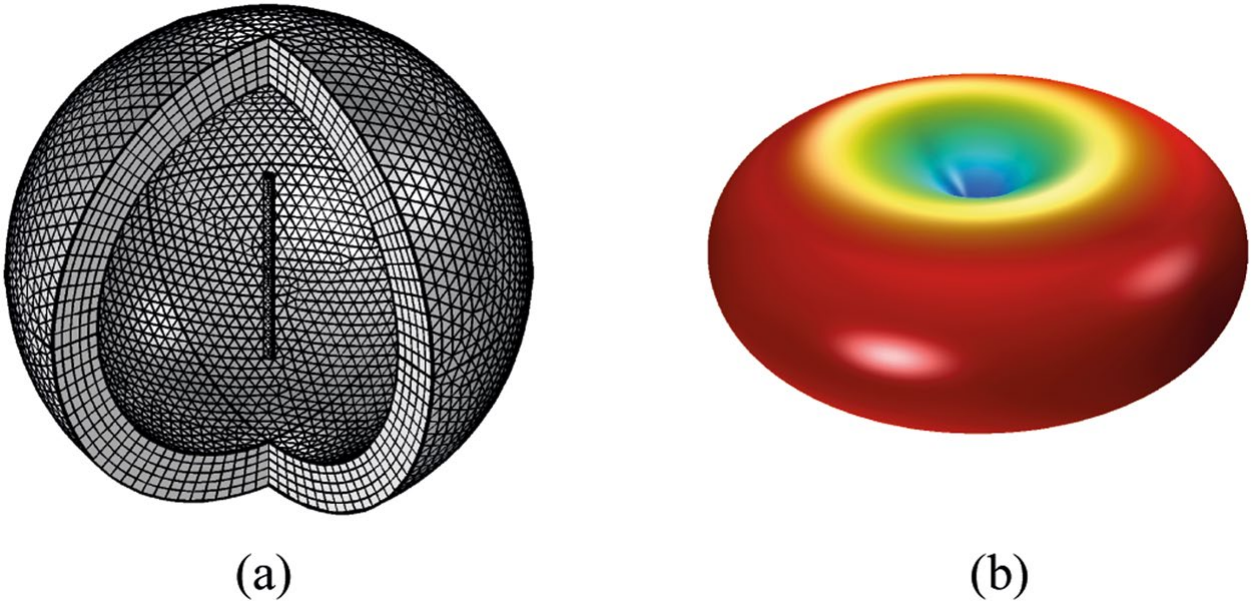
Single λ/4 antenna simulation where λ is 1 um. (**a**) Mesh of entire domain and dipole are shown. (**b**) the electric far field antenna pattern is shown.

The impedance boundary condition has been applied to take into account the correct approximation in the skin-depth. The impedance boundary condition is used at boundaries where the field is known to penetrate only a short distance outside the boundary. This penetration is approximated by a boundary condition to avoid the need to include another domain in the model. Although the equation is identical to the one in the low-reflecting boundary condition, it has a different interpretation. The material properties are for the domain outside the boundary and not inside, as for low-reflecting boundaries.

k_0_ is the wave number of free space ${k}_{0}=\omega \sqrt{{\mu }_{0}{\varepsilon }_{0}}$;

**E** is the electric field vector;

Figure 13 shows the simulation of a single λ/4 antenna able to radiate an electromagnetic field (1 µm wavelength). The dipole arm length is λ/4 (0.25 µm). The node radius is 0.5 µm. Voltage excitation at the lumped ports of each dipole is set to 100 mV. The frequency domain physics interface of FEM software allows the electric far field computation useful to plot the radiation pattern of antenna array.

**Figure 14:**
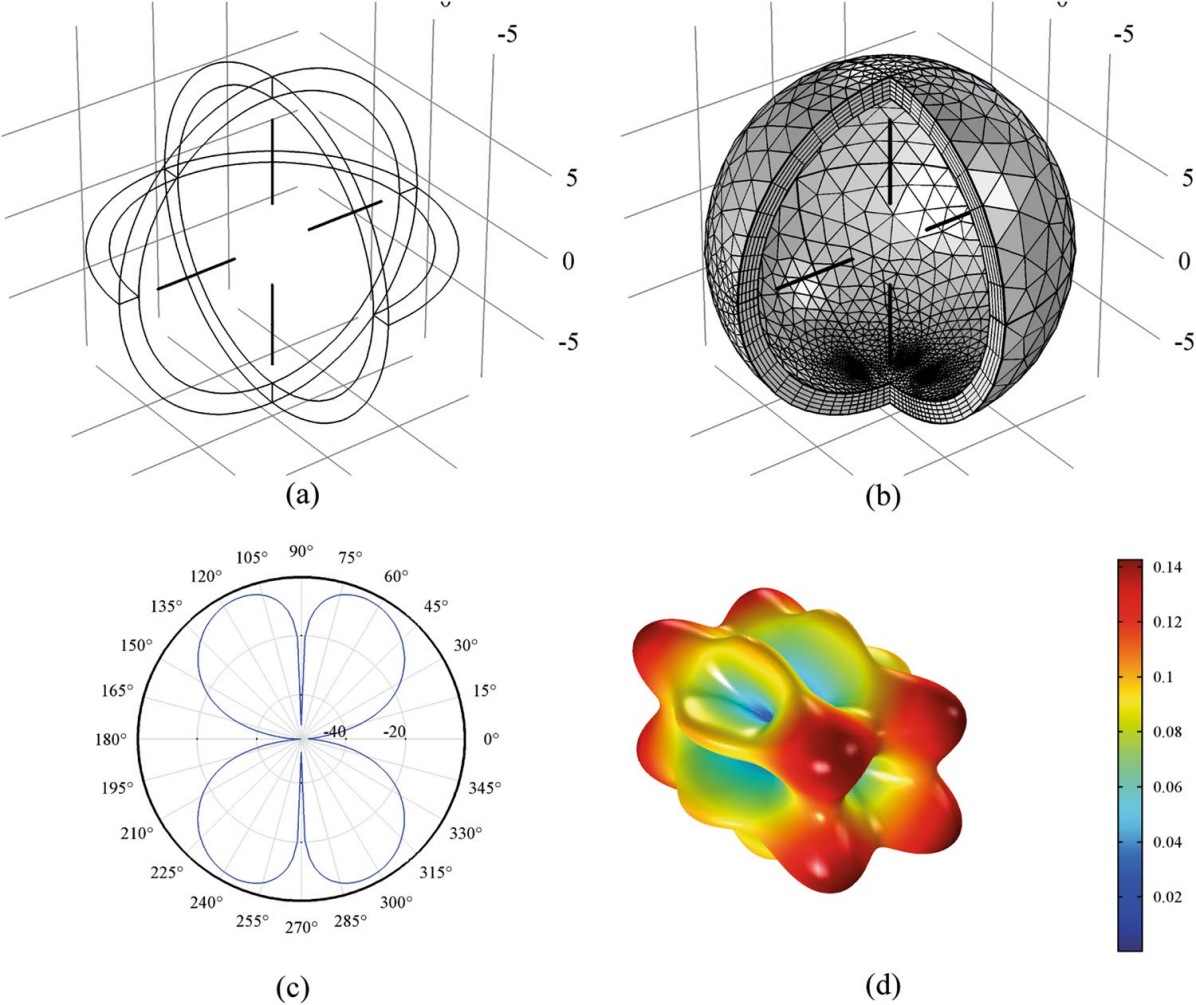
FEM simulation of λ/4 dipoles antenna array. (**a,b**) geometry and mesh of antenna array. (**c**) Antenna array far field gain dBi. (**d**) 3D pattern of the far electric field V/m.

Figure 14 show the simulation of four cross placed dipoles. This geometry provides an over-simplified scenario where only four ion channels, symmetrically placed, are the effective current sources in the elementary plane section of NR. In Fig. 14, the geometry, the mesh of the entire domain, the λ/4 dipole antennas and the computed electric far field pattern is shown. It is interesting to observe that even with such a simplified configuration, the higher values of electric far field are located along one coordinate direction, along the fiber axis and perpendicular to the plane section where dipole antennas are placed.

### Introduction to radiation pattern by Fortyλ/4 microdipoles antenna array

**Figure 15:**
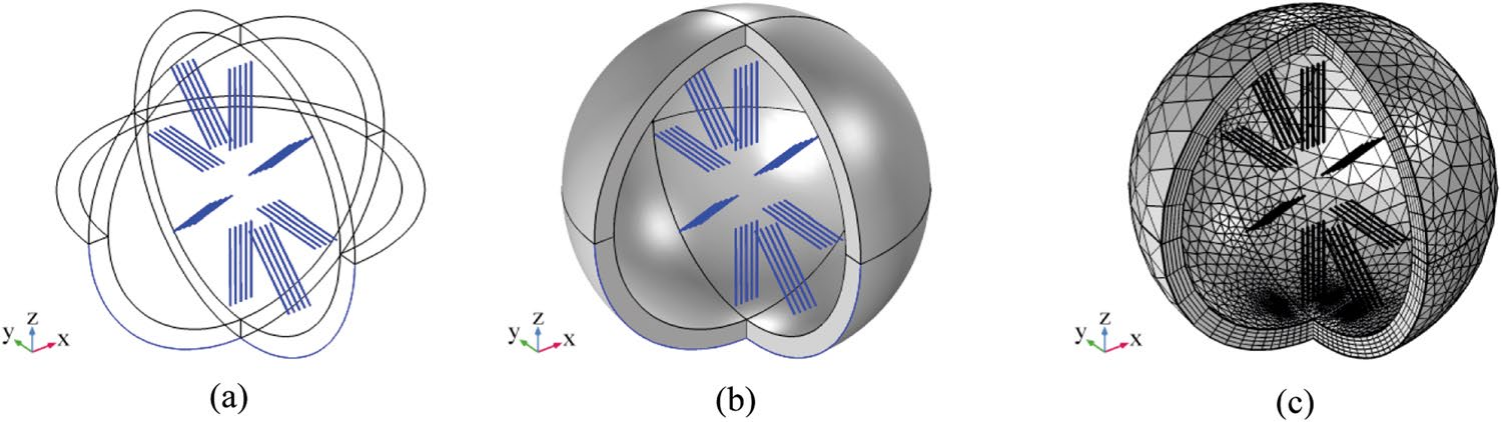
FEM simulation of forty λ/4 microdipoles at 1um of radiation wavelength. (**a,b**) geometry. (**c**) mesh of the 40 dipole antenna.

**Figure16:**
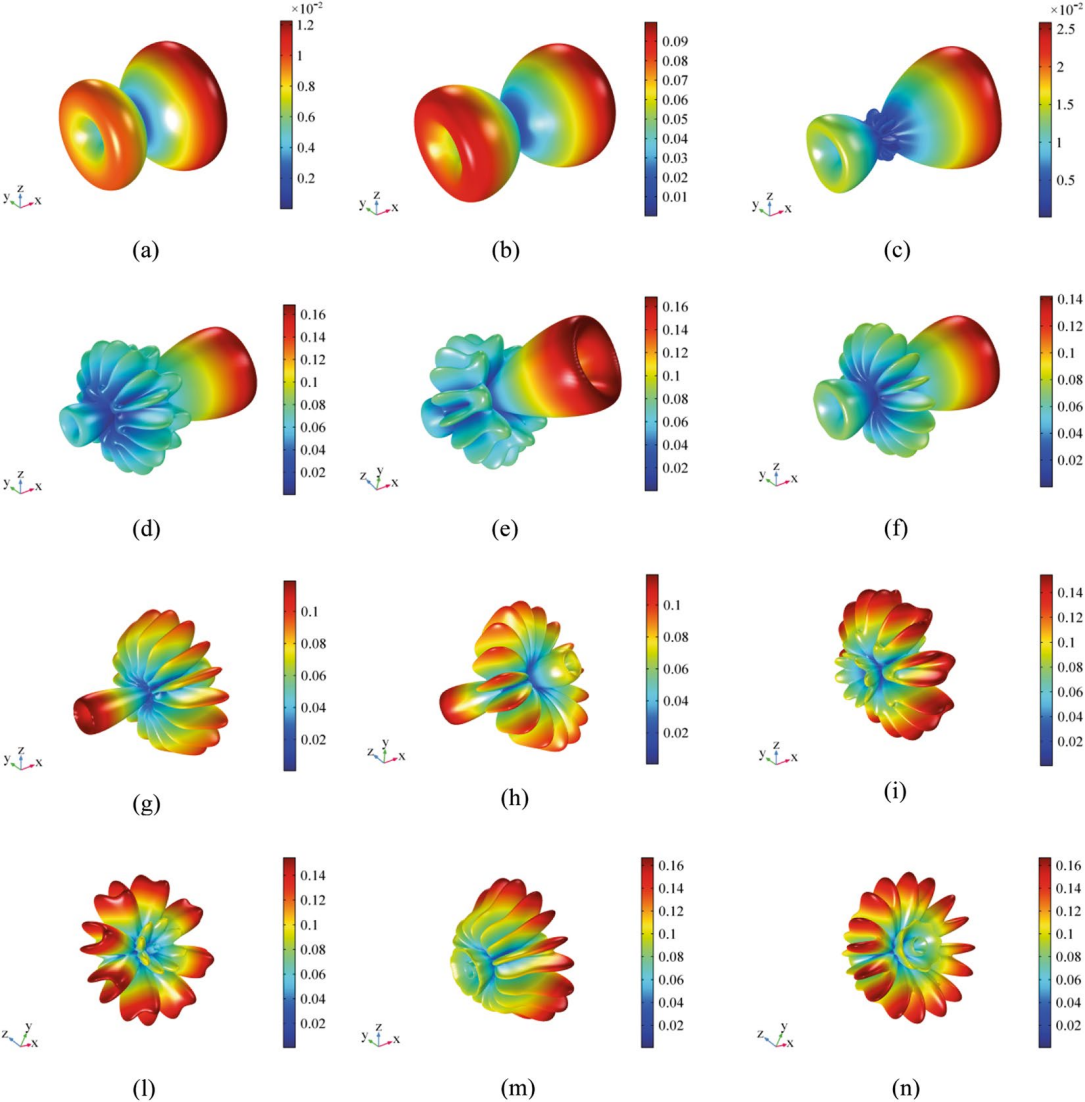
Electric far field V/m for different wavelength in the range 300–2500 nm. (**a**) 2.5 μm, (**b**) 1.2 μm, (**c**) 800 nm, (**d,e**) 600 nm, (**f**) 480 nm, (**g,h**) 400 nm, (**i**,**l**) 350 nm, (**m**,**n**) 300 nm.

Here the case of 40 λ/4 dipole antenna with λ = 1 µm is discussed. As before, the array consists of 5 planes, each containing 8 dipole antennas symmetrically placed around a node of radius 0.5 µm. In this full wave simulation, the voltage supply at the antenna ports is set to 100 mV and the feed phase delay time τ is set to 0.04 ns. Each plane of antennas is placed at a distance of 36 nm from the previous one as reported above in the results section^19^. Figure 15 shows the geometry, mesh, gain in dBi and far electric field expressed in V/m. Figure 16 shows the 3D far electric field pattern for different frequency values related to the wavelength range 300–2500 nm.

### Electromagnetic approach to myelinated axon

#### Finite Numerical Simulation Analysis of Rat Optic Nerve at Optic Wavelengths

For the FEM simulation the electromagnetic waves, beam envelopes (EWBE) physics modeling has been adopted. The EWBE is typically applied under the wave optics physics. It is used to compute electric and magnetic field distributions for systems and devices where the field amplitude varies slowly on a wavelength scale.

The physics interface can be used efficiently for unidirectional and bidirectional propagation of electromagnetic beams. With this physics interface, the electric field is factored into a product of an envelope function, which varies slowly over a wavelength and a rapidly varying phase function. The phase function is *a priori* prescribed, so the physics interface solves the time-harmonic wave equation for the slowly varying envelope function.

The electromagnetic studies have been computed applying the so called boundary mode analysis.

The boundary mode analysis study step is used to compute the propagation constants or wave numbers as well as propagating mode shapes, for a given wavelength at a port. As a study, the boundary mode analysis combines a boundary mode analysis study step at a port (boundary) (which can represent, for example, a cross section of a waveguide) with a frequency domain study step for the full geometry. The numerical solved equation is;

2 $$(\nabla -j{\bf{k}})\times {\mu }_{r}^{-1}((\nabla -j{\bf{k}})\times {\bf{E}})-{k}_{0}^{2}({\varepsilon }_{r}-\frac{j\sigma }{\omega {\varepsilon }_{0}}){\bf{E}}=0$$

where

ε_r_ is the real part of the relative electric permittivity obtained from the refraction index which is a known quantity;

μ_r_ is the is the real part of the relative magnetic permeability and is equal to 1 since no magnetic material are considered here;

σ is the electric conductivity and here is assumed equal to 0 because no losses are considered in this model;

**k** is the propagation wave vector here considered having the only component along the wave propagation direction i.e. longitudinal direction of the axon;

**E** is the electric field vector;

For the present ε_r_ and μ_r_ are assumed independent of the frequency. In these simulation the absorption or attenuation coefficients α (also called extinction coefficient), have been neglected but it is known that water (OH ions) cause the infrared absorption above 1.6 μm^30^. The propagation effective indexes^63^ found in this study are 0.980610753, 0.980613153, 0.984558112, 0.984560303, 1.0297277291318292, 1.029728359, 1.385934217, 1.385934218. The study runs over the frequency domain defined by the wavelength range 300–2500 nm.

In the FEM computation, the dimensioning and the automatic growth of the mesh have been optimized by setting the maximum element dimension to 1/10 of the electromagnetic field wavelength in the materials; this condition is essential to accurately solve the electric field in the Maxwell equations framework. To run the numerical simulation we provide a port input power of 10^−14^ W. The power at the port i.e. on the head of the dielectric waveguide (myelinated axon) could stay in a wide range of possibility, we think 10^−21^ ÷ 10^−10^ W, depending also by the useful head surface of myelinated axon effectively able to intercept the nanoantennas radiated field. This range has been hypostasized considering the electric far field of nanoantennas.

### Electromagnetic field analysis of the full structure of the node of Ranvier coupled to the myelinated axon through a paranode section

**Figure 17:**
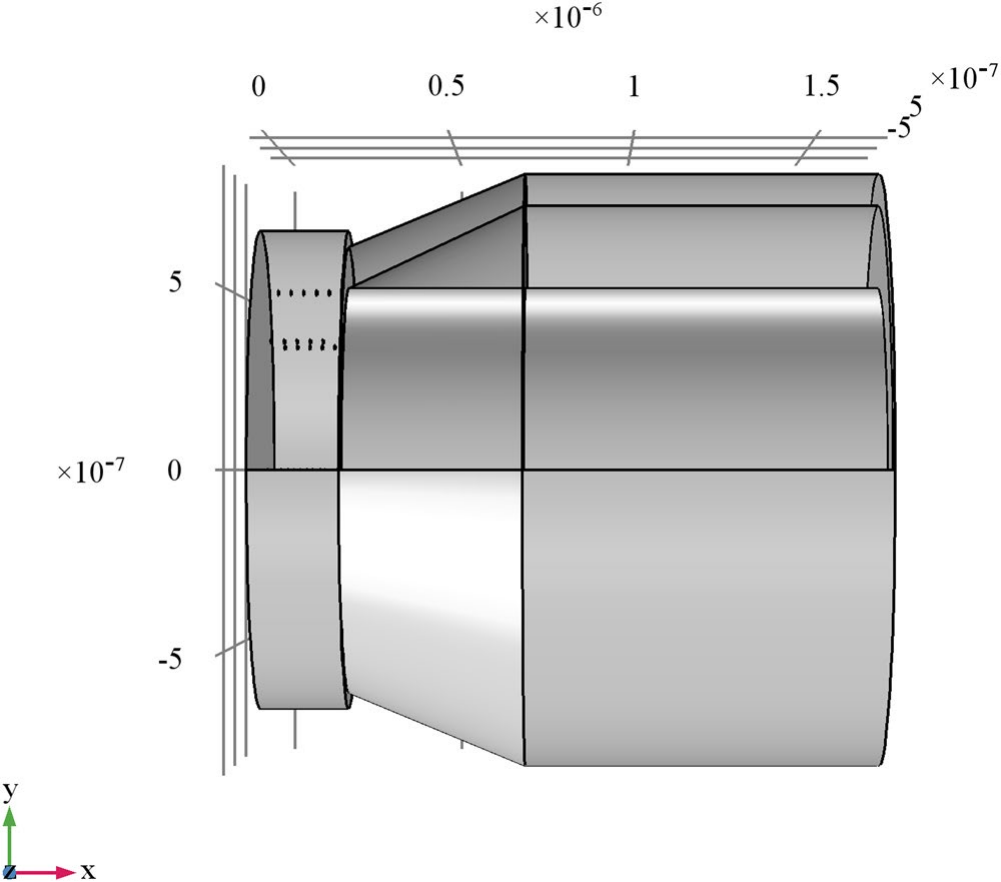
Geometry of the full structure of the node of Ranvier coupled to the myelinated axon through a paranode section. Dimensions of grid are in m.

**Figure 18:**
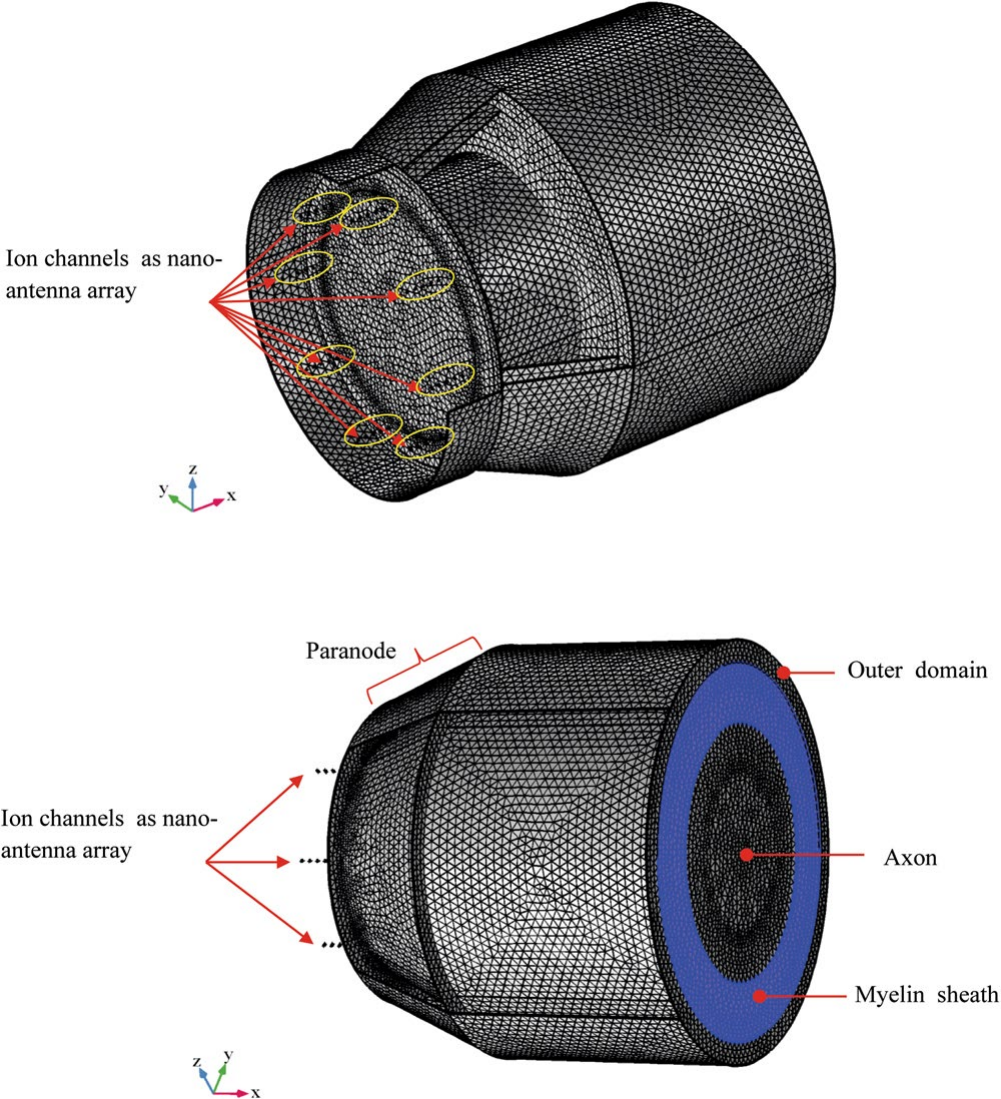
Mesh of the full structure of the node of Ranvier coupled to the myelinated axon through a paranode section.

In order to compute the electromagnetic field analysis, a full wave simulation is required. The equation (1) and the FEM method has been applied. The refraction index of each domain has been set in principle as in Fig. 4. In particular, in the annular paranode segment, it has been set equal to myelin layer. Compared to the scattered electromagnetic field reported in the previous section for nanoantennas array simulations, a full wave numerical simulation has been performed. These numerical simulations are very time and resources consuming. Such severe constraints limit the maximum length of axon segment to few microns. Nevertheless, the artificial nerve structure, though shorter than real, is long enough to show the propagation of electromagnetic field generated by the nanoantenna array through the paranode and myelinated axon structures. In order to compute the full electromagnetic wave numerical simulation, the geometry and the mesh of the entire structure have been provided. In Figs 17and 18 the geometry and mesh are shown. The mesh dimension has been chosen on the same basis as in the previous section. An outer domain layer surrounds all the paranode-myelinated axon geometry in order to take into account the extracellular medium.
